# Impact of the HOPE Intervention on Mental Health Literacy, Psychological Well-Being and Stress Levels amongst University Undergraduates: A Randomised Controlled Trial

**DOI:** 10.3390/ijerph19159772

**Published:** 2022-08-08

**Authors:** Jing Ling Tay, Yong Shian Shawn Goh, Kang Sim, Piyanee Klainin-Yobas

**Affiliations:** 1 West Region, Institute of Mental Health, Buangkok Green Medical Park, 10 Buangkok View, Singapore 539747, Singapore; 2 Alice Lee Centre for Nursing Studies, Yoo Loo Lin School of Medicine, 2 National University of Singapore Level 2, Clinical Research Centre, Block MD11, 10 Medical Drive, Singapore 117597, Singapore; 3 Yong Loo Lin School of Medicine, National University of Singapore, 10 Medical Drive, Singapore 117597, Singapore; 4 Lee Kong Chian School of Medicine, Nanyang Technological University, Clinical Sciences Building, 11 Mandalay Road, Level 18, Singapore 308232, Singapore

**Keywords:** mental health literacy, university students, depression literacy, anxiety literacy, stress, psychological well-being

## Abstract

Mental health literacy (MHL) promotes mental health among youths. We aimed to evaluate the effectiveness of the newly developed HOPE intervention in improving depression literacy, anxiety literacy, psychological well-being, and reducing personal stigma and stress levels amongst young adults at a university in Singapore. After two pilot studies, we conducted a randomised controlled trial (RCT) and recruited 174 participants aged 18–24 years old through social media platforms. The HOPE intervention group received four online sessions over two weeks and the control group received online inspirational quotes. Study outcomes were measured with self-reported questionnaires and they were assessed at baseline, post-intervention, and two-month follow-up (ClinicalTrials.gov: NCT04266119). Compared with the control arm, the intervention group was associated with increased depression and anxiety literacy levels at post-intervention and two-month follow-up. In addition, personal stigma for depression was reduced at the post-intervention juncture. However, there were no statistically significant changes in the ratings of psychological well-being and stress levels between the two groups. Longitudinal studies with larger sample sizes are warranted to replicate and extend the extant findings.

## 1. Introduction

Mental disorders (such as anxiety and depression) affect 20% of young adults globally and they impact emotional, psychological, and physical well-being and social functioning [1]. This situation causes serious concerns to mental health professionals as only one-third of those who experience mental disorders seek professional help [2]. Evidence suggests that individuals’ help-seeking attitudes can be enhanced with better mental health literacy [3]. In addition, anxiety and depression in young adults are linked with higher stress levels and poorer psychological well-being [4].

Stress refers to mental, emotional, and physical tensions experienced when individuals are unable to deal with threatening situations [5]. Youths experience various stressors related to their academic matters, interpersonal relationships, and socio-economic problems [1], elevating the risks of anxiety and depression. Psychological well-being (PWB) entails hedonic and eudaimonic aspects. The hedonic aspect focuses on maximising happiness and having positive rather than negative experiences [6,7]. The eudaimonic aspect concerns achieving happiness by attainment of self-actualisation or being a full-functioning person [7]. The eudaimonic component of PWB consists of environmental mastery, personal growth, self-acceptance, autonomy, having ideal and strong relationships, and a fruitful purpose in one’s life [8]. PWB is an essential factor in preventing mental disorders among youths and is positively correlated with emotional health recovery [9].

Mental health literacy (MHL) reflects individuals’ knowledge concerning specific mental disorders, which helps them to prevent, identify or manage the disorders [10]. Therefore, MHL is crucial in primary care settings to facilitate prompt identification, treatment, and recovery of mental disorders [11]. In addition, MHL can be enhanced through educational programs disseminated via information and communication technology (ICT) using telecommunication devices such as robots, smartphones, computers, and emails to deliver digital knowledge.

Existing ICT-MHL interventions (such as Beyond Blue and MoodGym) have been tested more in European countries and Australia but less seen in Asia [12,13]. Thus, in this study, we developed the HOPE intervention, an ICT-MHL intervention, specifically for young adults in Singapore. The HOPE intervention comprises a combination of relaxation exercises, positive psychology techniques, and cognitive-behavioural exercises, which appeared to reduce stress and enhance psychological well-being [14]. Findings from our two pilot studies suggested positive effects of the HOPE intervention [15]. In this paper, we aimed to evaluate the effectiveness of the HOPE intervention in enhancing depression and anxiety literacy, and PWB; and reducing stigma and stress among young people living in Singapore.

## 2. Methods

### 2.1. Study Design

The study is a two-armed, parallel, randomised controlled trial. Participants were randomly assigned to either an intervention or control group. Online participant recruitment period was from 16 February 2020 to 27 February 2020. The recruitment was conducted without face-to-face interaction due to infection control measures during the COVID-19 pandemic. An independent member of staff generated a random allocation list via computer software before recruitment. Allocation concealment was fulfilled with sealed and opaque envelopes. After baseline assessment, the participants were assigned to their groups. Due to the nature of this study, participants were not blinded to the group allocation.

### 2.2. Participants

Convenience sampling was used to recruit participants at a university in Singapore. Inclusion criteria were young adults: (a) aged 18 to 24 years old with internet access and (b) were literate in English. Exclusion criteria were participants with reading or hearing problems. Eligible participants were recruited from the university’s social media channels, including Twitter, Telegram, Facebook, Instagram, and SnapChat. Details of the study and an invitation to participate were posted on these social media. Interested participants were asked to contact the researchers using their university email. The email also served to identify participants as students of the same university. Patients and public were not involved in the research study’s conduct, design, or report.

### 2.3. Sample Size Calculation

A study conducted in Australia that evaluated the effectiveness of a website intervention on help-seeking intentions had an effect size of 0.53 [13]. With this effect size (d = 0.53), probability level of 0.05, and power of 0.8, the required sample size would be at least 57 participants per group [16]. Considering the possible high drop-out rate of 70% [17], the sample size was set at 97 per group.

### 2.4. Control Group

Participants in this group were asked to log on to a website containing inspirational quotes such as, “Today is full of possible” and “You can do anything”. The participants were required to review the website at least once during the study.

### 2.5. Intervention Group

In addition to the website with inspirational quotes, the intervention group received HOPE intervention [15]. The authors designed the HOPE intervention based on extant empirical evidence [18], suggesting that ICT-MHL interventions using case studies, quizzes, and videos elevated depression literacy [13,17,18,19]. The contents of the HOPE intervention included myths, causes, clinical symptoms, non-pharmacological and pharmacological management of depression and anxiety disorders [13,17,19,20,21]. The intervention protocol was reviewed by three clinicians using a content validity evaluation tool [22]. An average intraclass correlation of 0.97 with a 95% confidence interval from 0.9 to 1 [F (2, 166) = 36.55, *p* < 0.001), indicated high agreement among the three clinicians [22].

Two pilot studies were conducted to evaluate the initial outcomes of the HOPE intervention. Results of the first pilot study revealed high drop-outs and poor adherence to the intervention. Hence, a second pilot study was conducted with the following changes based on feedback from the first pilot study: (1) shortened questionnaires, (2) weekly WhatsApp and email reminders, (3) incentives for participants who accessed the intervention, and (4) shortened duration of the intervention (from four to two weeks). Results from the second pilot study revealed that the research design and revised procedures were more acceptable to the participants.

The HOPE intervention consisted of four sessions. Participants were asked to attend two sessions per week for two weeks. Each session lasted about ten minutes and contained pre-post quizzes, video(s), and illustrations about mental health. Session one comprised information on myths, symptoms, causes of depression, self-help strategies, and treatments for depression. Session two included practical examples of positive psychology such as expressions of gratitude, affect, and strength-based exercises. Session three covered information regarding myths, causes, symptoms, self-help strategies, and treatments of anxiety disorders. Session four encompassed relaxation exercises and self-management strategies for unhealthy thoughts that utilized techniques from cognitive behavior therapy. Weekly reminders through emails and WhatsApp were disseminated to participants to complete their sessions.

### 2.6. Measurements

The Depression Literacy Questionnaire (D-Lit) was used to measure depression literacy [23,24]. The D-Lit consists of 22 questions scored ‘don’t know’, ‘true’, and ‘false’. Every correct response will score a point. Possible scores ranged from 0 to 22, with higher scores indicating greater depression literacy. Among athletes, an alpha coefficient of 0.70, and test re-test reliability of 0.71 were reported for the D-Lit [21]. The tool was used in a previous local study [24].

The Anxiety Literacy Questionnaire (A-Lit) was used to assess anxiety literacy levels amongst participants [21]. The tool consists of 22 questions scored ‘don’t know’, ‘true’, and ‘false’. Every correct response will score a point, with a maximum score of 22. Among athletes, the reliability statistic was 0.76, and test re-test reliability was 0.83 [21]. In this RCT study, A-Lit achieved a Cronbach’s Alpha of 0.71.

The Personal Stigma Scale consists of two subscales: ‘unpredictable/dangerousness’ and ‘weak, not sick’ [25,26]. Each question was scored from 5 ‘strongly agree’ to 1 ‘strongly disagree’, with higher scores implying more significant stigmatising attitudes. The scale was used in a previous local survey [26] and confirmatory Factor Analysis revealed an acceptable construct validity of the scale amongst the people in Singapore [26].

The Psychological Well-being Scale (18 items) was used to measure psychological well-being, comprising six domains: autonomy, personal growth, environmental mastery, self-acceptance, good social relationships, and life purpose [8,27]. The responses for the questions were from 1 strongly agree to 7 strongly disagree. The score ranged from 18 to 126 with greater scores equate to higher PWB. Among 321 adult Americans, test re-test reliability coefficients over six weeks were 0.88 (autonomy), 0.85 (self-acceptance), 0.81 (personal growth), 0.81 (environmental mastery), 0.82 (life purpose) and 0.83 (positive social relationships) [8]. The Psychological Well-being Scale was associated with domains of ideal functioning (e.g., morale, internal control, life happiness, self-esteem, and affect balance), and the coefficients were 0.25–0.73, thus establishing convergent validity. The scale used in a local study amongst university and polytechnic students achieved a Cronbach’s alpha of 0.84 [27].

The Perceived Stress Scale (PSS) measures stress levels [28]. The ten questions scale ranges from 0 never to 4 very often. The scale consists of four positively-worded items with reversed scoring [28]. Scoring ranged from 0 to 56, with higher scoring equating to higher stress levels. The convergent validity of the scale was established with its significant association with depression, anxiety, and physical symptoms [28]. The scale had a reliability coefficient of 0.78 [29]. The PSS was used in a study among 200 undergraduates in Singapore [30]. After excluding item 7, the coefficient alpha was 0.85 [30]. In another study conducted amongst students in Singapore, the scale achieved a Cronbach’s Alpha of 0.89 [27].

### 2.7. Data Collection Procedure

All participants completed a baseline assessment online. After that, participants were randomly assigned to 2 groups and were given access to either control or HOPE intervention according to the allocation. In the control group, participants were told to log onto an online website to view its contents at least once during the study. The HOPE intervention group received weekly email reminders to complete the intervention sessions for two weeks. They were invited to read the website’s contents, watch the videos, and complete each session’s pre- and post-quizzes. Participants who missed the session received a second reminder to complete the session. Next, data collection using online questionnaires was conducted at the post-intervention time-point and two-month follow-up. All online questionnaires were accessed through a unique URL link sent to the university email address of each individual participant.

### 2.8. Data Analyses

All statistical analyses were performed using SPSS Version 22.0. The normality of quantitative data was checked using the Shapiro-Wilks test, histograms, skewness, kurtosis, and Q-Q plots. Differences between groups were tested by *t*-test and Mann-Whitney U-test for normal and non-normal continuous variables, respectively, and Chi-square test or Fisher exact test for categorical variables whenever appropriate. The repeated measure ANOVA was used to examine the changes in outcome measures at different time points, and paired *t*-tests were used to examine the differences between both groups at each time point. All *p* values were two-tailed at the significance level of *p* < 0.05. The research was approved by the Institutional Review Board of the National University of Singapore (S-19-251). The study was registered at ClinicalTrial.gov with the identifier NCT04266119.

### 2.9. Patient and Public Involvement

There was no patient involvement. Undergraduate students from the University were participants in this study.

## 3. Results

**Figure 1:**
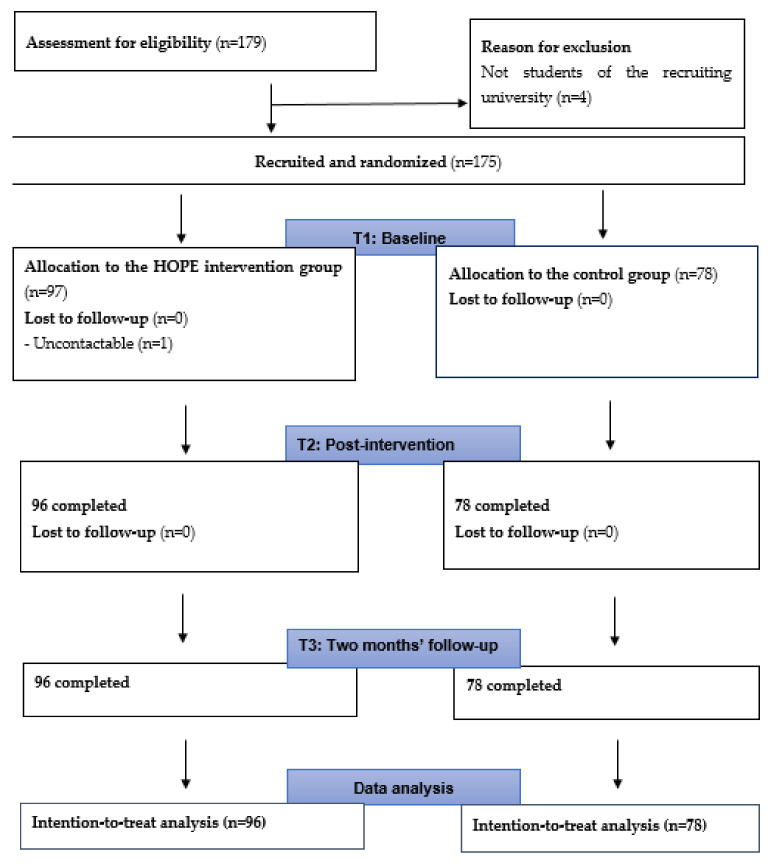
Consort flowchart.

We recruited 179 participants via various social media platforms within two weeks in February 2020. A total of 174 participants completed all the questionnaires at the three assessment points. Please refer to Figure 1 for the details of subject flow in the two arms and Table 1 for characteristics of the participants. One participant dropped out after indicating his interest in participating in the research study. He did not complete any questionnaire. The online statistic tracker showed that all participants in both groups attended the website with inspirational quotes. In addition, all participants in the intervention group participated all four HOPE sessions.

### 3.1. Effects of the HOPE Interventions on Outcomes

At baseline, neither group had significant differences in the demographic features, MHL levels, stress, and PWB scores.

#### 3.1.1. Depression Literacy

The main effect of the group on depression literacy was statistically significant [F (1, 172) = 13.67, *p* <0.001]. The time and group interaction was statistically significant [F (2, 344) = 44.22, *p* < 0.001] with a small to medium effect size (Partial η_p_^2^ = 0.21) (Table 2). Compared with the control group, the intervention group had significantly higher depression literacy at post-intervention (*t =* −6.15, *p* < 0.001) and at two-month follow-up (*t* = −4.66, *p* < 0.001) (Table 3).

#### 3.1.2. Anxiety Literacy

The main effect of the online intervention on anxiety literacy was statistically significant [F (1, 172) = 17.18, *p* < 0.001]. The time and group interaction was statistically significant [F (1.76, 303.09) = 29.06, *p* < 0.001] with a small effect size (η_p_^2^ = 0.15) (Table 2). Compared with the control group, the intervention group had significantly higher anxiety literacy at post-intervention (*t* = −6.28, *p* < 0.001) and at two-month follow-up (*t* = 4.54, *p* < 0.001) (Table 3).

#### 3.1.3. Personal Stigma (Depression)

The main effect of group on personal stigma (depression) was statistically non-significant, [F (1, 172) = 0.73, *p* = 0.4, η_p_^2^ = 0.004]. The time and group interaction was statistically significant, [F (2, 344) = 11.08, *p* < 0.001]. Overall, the HOPE intervention had a statistically significant effect on personal stigma for depression post-intervention (*t* = −2.26, *p* = 0.03). At two months’ follow-up, although the finding was not statistically significant, the participants in the HOPE intervention had lower levels of personal stigma (refer to Table 3).

#### 3.1.4. Stress and Psychological Well-Being

There were no statistically significant changes in stress and psychological well-being levels between and within groups. At both post-intervention and two-month follow-up, participants in the HOPE intervention group had lower stress levels than participants in the control group, but this did not reach statistical significance.

### 3.2. Factors Influencing Depression and Anxiety Literacy

Additional analyses (Table 4) revealed that gender, the discipline of study, and previous experiences with mental healthcare staff influenced baseline literacy scores. Specifically, female students (M = 10.85, SD = 3.45) had a higher level of anxiety literacy (F = 6.75, *p* = 0.01) than males (M = 9.32, SD = 3.64). In addition, participants from healthcare-related disciplines such as psychology (*n* = 16), medicine (*n* = 6), pharmacy (*n* = 5), and nursing (*n* = 1) had higher depression and anxiety literacy scores.

In terms of depression literacy, psychology students scored the highest (M = 15.06, SD = 3.34, *n* = 16) followed by one student each from nursing and integrative sciences and engineering courses (M = 15, *n* = 1), medical (M = 14, SD = 2.76, *n* = 6), and pharmacy students (M = 14, SD = 2.45, *n* = 5). In terms of anxiety literacy, the nursing student scored the highest (M = 17, *n* = 1), followed by medical (M = 14.33, SD = 2.73, *n* = 6) and psychology students (M = 12.88, SD = 3.91, *n* = 16). Students from the Faculty of Engineering scored the lowest (M = 9.16, SD = 3.09), followed by students from Business (M = 9.62, SD = 3.15) and Arts/Social Sciences schools (M = 9.78, SD = 3.06).

Participants with previous contact with mental health professionals had different levels of depression literacy (M = 12.05, SD = 3.41; F = 4.25, *p* = 0.003) and anxiety literacy (M = 10.41, SD = 3.57; F = 8.33, *p* ≤ 0.001) as compared to participants without the contact. Generally, participants with prior contact with mental healthcare professionals had higher depression and anxiety literacy scores.

Participants who knew healthcare professionals had the highest levels of depression literacy (mean = 13.47, SD = 3.54, *n* = 34) and anxiety literacy (mean = 12.21, SD = 3.63, *n* = 34). Participants who did not know any healthcare professional had the lowest depression literacy (mean = 11.47, SD = 3.31, *n* = 122) and anxiety literacy levels (mean = 9.61, SD = 3.35, *n* = 122).

Other factors such as ethnicity, religion, residential status, personal experiences of mental health problems, and having families/friends with psychiatric problems were not associated with depression and anxiety literacy levels.

## 4. Discussion

Several main findings were noted in this study. First, participants in the HOPE intervention group had higher depression and anxiety literacy scores at post-intervention and two-month follow-up. Second, personal stigma for depression was reduced at the post-intervention juncture. Third, there were no statistically significant changes in stress and PWB levels following the HOPE intervention.

### 4.1. Depression Literacy

The intervention group had higher depression literacy scores at post-intervention and two-month follow-up. This was likely attributed to session one of the HOPE intervention, which depicted depression in detail. Our findings were congruent with existing studies, whereby participants in intervention groups reported increased depression literacy scores after participation [12,17,20,21,31,32,33]. The interventions included mental health first aid course [20], educational PowerPoint slides about autism [31], 34 web pages about depression [21], Ching Ching Story [17], and a four-minute informational section about anorexia [12]. In terms of content, the HOPE intervention had comparable components to those previous interventions in educating the subjects on a wide range of areas, including myths, prevalence, symptoms, risk factors, causes, and treatments of specific mental disorders.

### 4.2. Factors Affecting Depression Literacy

Females did not differ in depression literacy scores compared with males. This was consistent with one study [34]; nevertheless other studies found that females reported higher depression literacy scores than males [35,36,37,38,39,40]. A possible explanation could be that females tend to possess greater insight on their health and have more concern with their emotional well-being. They tend to verbalize more about depressive symptoms than males [41]. To date, one study reported that males have greater MHL regarding mental disorders [42].

### 4.3. Anxiety Literacy

Participants who received the HOPE intervention had higher anxiety literacy scores at post-intervention and two-month follow-up compared to the control group. This may be attributable to session three of the HOPE intervention, which specifically described anxiety disorders in detail. Similar findings were reported from another study that used the medium of 34 web pages among elite athletes [21]. The 34 web pages covered risks, prevalence, symptoms, treatments, and myths regarding anxiety disorders that were comparable with the themes covered in the HOPE intervention. In addition, the HOPE intervention included videos and quizzes that might enhance the participants’ learning.

### 4.4. Factors Affecting Anxiety Literacy

Females have higher anxiety literacy scores compared with males. In a UK study of 317 adult participants, females were more likely to correctly identify anxiety disorders, post-traumatic stress disorder, obsessive-compulsive disorder, and specific phobia than males [43]. In contrast, in another study of 270 university undergraduates in the USA, females were found to under-identify social anxiety disorder and generalized anxiety disorder [44]. However, another study amongst 1104 US high school students found no gender differences in recognition of social phobia [36].

In terms of discipline, healthcare undergraduates in nursing, psychology, and pharmacy were associated with higher depression and anxiety literacy scores. Conversely, students from non-healthcare undergraduate fields (such as business and engineering) had lower depression and anxiety literacy scores. Our findings were comparable with the observations of earlier studies [34,35,38]. Similarly, undergraduates [34] and postgraduate students [45] reported higher depression literacy scores, suggesting that exposure and familiarity with a curriculum covering healthcare-related topics might help raise literacy about mental disorders. Our additional finding of the association of “contact with mental healthcare professional” with higher depression and anxiety literacy scores also supported this notion.

Although contact with mental health professionals presumably in the course of study was associated with better literacy levels, direct or indirect experiences with mental disorders, either personally or through close ones (families and friends), were not associated with depression and anxiety literacy scores. This was in agreement with other studies which reported no difference in depression literacy scores between participants with or without personal experiences of depression [34,38,46] and participants with or without family members with depression [34]. Of note, a previous review found that the public members who had contact with those experiencing psychiatric symptoms were better at recognizing these disorders [47]. A study by Paulus et al. [44] found that participants with mental disorders tended to under-rate mild to moderate anxiety cases and over-rate severe cases of anxiety disorders.

### 4.5. Personal Stigma (Depression)

Participants who received the HOPE intervention had a lower personal stigma about depression post-intervention than the control group; however, this result was not sustained at the two-month follow-up. The improvement of stigma post-intervention was likely associated with session one of the HOPE intervention, which covered the prevalence and myths regarding depression. An additional video clip showed a character with symptoms of depression who eventually sought help for his distress. Taken together, these elements of the HOPE intervention may have helped to reduce personal stigma following the intervention. This finding was congruent with earlier findings that mental health first aid courses and contact videos reduced stigma [20,48]. The online HOPE intervention could be enhanced by incorporating video narratives of people sharing their struggles with mental disorders to further reduce stigma [48].

### 4.6. Stress and Psychological Well-Being

The HOPE intervention reduced stress at post-intervention and two-month follow-up, but this did not reach statistical significance. Furthermore, the HOPE intervention did not improve psychological well-being. The HOPE intervention included positive psychology exercises, relaxation exercises, and self-management of unhelpful thoughts. These exercises were thought to enhance the participants’ coping strategies in managing their stress levels [14,49,50,51]. There are possible reasons to account for the non-significant findings. First, the focus of the HOPE intervention was to enhance MHL, and the taught exercises to reduce stress were a small portion of the intervention. Second, the adherence and practice duration of these exercises were not monitored; hence, it was difficult to determine whether participants were practising what they had learned. Third, the videos that specifically addressed these exercises in sessions two and four of the online HOPE intervention were long in duration. This might discourage the participants from reviewing the entire video [52]. Fourth, compared to information about mental disorders, psychological techniques take time to understand and internalize. Lastly, the study was likely under-powered to show statistically significant changes in PWB and stress levels.

## 5. Limitations

This study has a few limitations. First, the participants were recruited from a single site in a university setting, which limits the generalizability to other groups. Second, this study used self-reported questionnaires, which might lead to a social desirability bias. Third, the follow-up assessment was performed up to two months later and therefore changes in literacy levels beyond this period might not be captured. Finally, the study did not capture participants’ adherence to positive psychology and relaxation exercises.

## 6. Contributions of the Study

The study addressed the current knowledge gap, as there was a relative paucity of research about mental health literacy interventions in Asia. This is the first randomised controlled trial that developed and tested the efficacy of the online HOPE program in improving MHL in Singapore. The study also added new findings in terms of associated intrapersonal factors associated with depression and anxiety literacy. This study emphasized the importance of mental health education in healthcare settings, particularly primary care settings. With the availability of MHL interventions, youths can seek help regarding their mental health concerns if needed. Our findings may encourage better mental health education for the public and technology could be harnessed to disseminate MHL interventions more extensively and expeditiously.

## 7. Implications

The online HOPE intervention could be extended to a variety of groups of individuals, including: (1) youths already diagnosed with mental disorders, (2) youths at risk for developing mental disorders, (3) their caregivers, (4) their classmates, (5) school counsellors and school teachers. Future studies can evaluate the efficacy of the online HOPE intervention within these groups. This can potentially facilitate earlier help-seeking, treatment, and recovery, eventually reducing the illness burden over time.

## 8. Future Research Directions

The findings of this study can be replicated in studies involving young adults in various settings. Longitudinal studies can be conducted to examine the effects of MHL changes on psychological well-being, depression, and anxiety levels to ascertain actual clinical benefits over different periods. Longitudinal studies can also examine the impact and sustainability of such MHL interventions. Additionally, qualitative research can help elicit findings to complement the quantitative results when evaluating the participants’ perspectives on the interventions. As this study was conducted during the COVID-19 pandemic, future research could also examine the students’ psychological well-being and stress levels after the COVID-19 pandemic.

## 9. Conclusions

In conclusion, the HOPE intervention was associated with increased MHL scores (anxiety and depression) and reduced personal stigma regarding depression among local university students. However, further studies involving longer follow-up, larger sample populations, and incorporating qualitative feedback are warranted to replicate and extend the extant findings.

## Figures and Tables

**Table 1:** Baseline demographic features and rating scores of the subjects within the 2 arms.

	Intervention (*n* = 96)	Control (*n* = 78)	*χ* ^2^	*p*
	Frequency (%)	Frequency (%)		
Age			2.83	0.09
18–21	64 (66.67%)	61 (78.21%)		
22–24	32 (33.33%)	17 (21.79%)		
Gender			0.02	0.89
Male	28 (29.17%)	22 (28.21%)		
Female	68 (70.83%)	56 (71.79%)		
Residential status			2.56	0.28
Singaporean	90 (93.75%)	68 (87.18%)		
Permanent resident	2 (2.08%)	2 (2.56%)		
Student pass	4 (4.17%)	8 (10.26%)		
Ethnicity			1.10	0.78
Chinese	87 (90.63%)	68 (87.18%)		
Malay	2 (2.08%)	1 (1.28%)		
Indian	6 (6.25%)	8 (10.26%)		
Others	1 (1.04%)	1 (1.28%)		
Religion			7.10	0.42
Christian	16 (16.67%)	19 (24.36%)		
Catholic	9 (9.34%)	3 (3.85%)		
Buddhist	25 (26.04%)	18 (23.08%)		
Taoism	8 (8.33%)	2 (2.56%)		
Muslim	2 (2.08%)	3 (3.85%)		
Hindu	4 (4.17%)	4 (5.13%)		
Free-thinker	28 (29.17%)	27 (34.62%)		
Others	4 (4.17%)	2 (2.56%)		
Family/friends with mental health problems			0.50	0.78
Yes	70 (72.92%)	58 (74.36%)		
No	13 (13.54%)	8 (10.26%)		
Unsure	13 (13.54%)	12 (15.38%)		
Personal experiences with mental health problems			3.31	0.35
Yes	8 (8.33%)	8 (10.26%)		
No	62 (64.58%)	44 (56.41%)		
Not sure did not seek help	26 (27.08%)	24 (30.77%)		
Others	0	2 (2.56%)		
Contact with mental health professionals			2.43	0.66
Yes, for myself	10 (10.42%)	12 (15.38%)		
Yes, for my friends/family	10 (10.42%)	4 (5.13%)		
No	67 (69.79%)	55 (70.51%)		
Yes, I know them personally	7 (7.29%)	5 (6.41%)		
Others	2 (2.08%)	2 (2.56%)		
	**Mean (SD)**	**Mean (SD**)	***t***	***p***
Depression literacy	11.8 (3.59)	12.36 (3.17)	1.07	0.29
Anxiety literacy	10.39 (3.83)	10.44 (3.23)	0.09	0.93
Personal stigma (depression)	16.55 (3.99)	15.77 (3.79)	−1.31	0.19
PWB	86.64 (16.24)	88.40 (13.78)	0.76	0.45
Stress	20.19 (5.69)	20.10 (6.43)	−0.09	0.93

*p* = *p* value, SD = Standard deviation, *t* = *t* statistic, *χ*^2^ = Chi square.

**Table 2:** Comparison of outcomes (depression literacy, anxiety literacy, personal stigma, stress, psychological well-being) between 2 groups over time.

	Effects	Type III Sum of Squares	df	F	*p* Value	η_p_^2^
**Depression literacy**	Group	384.12	1	13.67	<0.001	0.07
	Time-points	263.29	2	33.67	<0.001	0.16
	Group × Time	345.77	2	44.22	<0.001	0.21
**Anxiety literacy**	Group	519.87	1	17.18	<0.001	0.09
	Time-points	365.22	1.76	36.93	<0.001	0.18
	Group × Time	287.33	1.76	29.06	<0.001	0.15
**Personal stigma**	Group	25.83	1	0.73	0.4	0.004
	Time-points	47.46	2	5.05	0.007	0.028
	Group × Time	104.24	2	11.08	<0.001	0.061
**Stress**	Group	22.44	1	0.23	0.63	0.001
	Time-points	0.56	2	0.03	0.97	0
	Group × Time	22.63	2	1.14	0.32	0.007
**Psychological well-being**	Group	256.28	1	0.4	0.53	0.002
	Time-points	9.75	2	0.15	0.86	0.001
	Group × Time	64.64	2	0.98	0.38	0.006

Df = degree of freedom, F = F statistic, η_p_^2^ = Partial eta Square.

**Table 3:** Comparisons of depression and anxiety literacy, stigma, psychological well-being, and stress scores between the two groups.

		Intervention		Control					
	Time-Points	*n*	M (SD)	*n*	M (SD)	Mean Difference	*t*	95% CI	*p* Value
Depression literacy	Baseline	96	11.80 (3.59)	78	12.36 (3.17)	−0.56	−1.07	[−1.58–0.47]	0.29
	Post-intervention	96	15.24 (3.55)	78	12.04 (3.24)	3.2	6.15	[2.17–4.23]	<0.001
	2-month follow-up	96	14.81 (3.86)	78	12.28 (3.15)	2.53	4.66	[1.46–3.60]	<0.001
Anxiety literacy	Baseline	96	10.39 (3.83)	78	10.44 (3.23)	−0.05	−0.09	[−1.13–1.03]	0.93
	Post-intervention	96	13.98 (3.94)	78	10.54 (3.12)	3.44	6.28	[2.36–4.52]	<0.001
	2-month follow-up	96	13.44 (4.05)	78	10.81 (3.46)	2.63	4.54	[1.49–3.77]	<0.001
Personal stigma	Baseline	96	16.55 (3.99)	78	15.77 (3.79)	0.78	1.31	[−0.39–1.96]	0.19
	Post-intervention	96	14.75 (3.67)	78	16.09 (4.17)	−1.34	−2.26	[−2.52–0.17]	0.03
	2-month follow-up	96	15.44 (3.92)	78	16.22 (3.68)	−0.78	−1.34	[−1.93–0.37]	0.18
Stress	Baseline	96	20.19 (5.69)	78	20.10 (6.43)	0.08	0.09	[−1.73–1.90]	0.93
	Post-intervention	96	19.94 (6.10)	78	20.33 (6.73)	−0.40	−0.41	[−2.32–1.53]	0.69
	2-month follow-up	96	19.74 (6.48)	78	20.68 (6.30)	−0.94	−0.96	[−2.87–0.99]	0.34
Psychological well-being	Baseline	96	86.64 (16.24)	78	88.40 (13.78)	−1.76	−0.76	[−6.33–2.81]	0.45
	Post-intervention	96	87.27 (15.25)	78	87.69 (16.13)	−0.42	−0.18	[−5.13–4.29]	0.86
	2-month follow-up	96	86.19 (16.18)	78	88.23 (14.16)	−2.04	−0.88	[−6.65–2.56]	0.38

M = Mean, SD = Standard deviation, *t* = *t* statistic.

**Table 4:** Relationship between participants’ characteristics and depression and anxiety literacy scores.

Participants’ Characteristics	D-Lit		A-Lit	
	F	*p*	F	*p*
s	1.03	0.31	6.75	* 0.01
Ethnicity	2.17	0.93	0.29	0.83
Religion	1.03	0.42	0.78	0.6
Course of study	2.18	* 0.02	3.01	** 0.001
Monthly household income	0.16	0.99	0.2	0.98
Satisfaction with monthly household income	0.75	0.59	1.36	0.24
Family/friends with mental health problems	2.57	0.08	1.55	0.22
Contact with mental healthcare professional	4.25	** 0.003	8.33	** <0.001
	Welch	*p*	Welch	*p*
Age	1.84	0.18	2.94	0.09
Residential status	0.03	0.97	0.72	0.52
Personal experiences with mental health problems	3.36	0.11	3.43	0.11

F = F statistic, *p* = *p* value, * *p* < 0.05, ** *p* < 0.01.

## Data Availability

The protocol of the study can be requested via email to jing_ling_tay@imh.com.sg. With reasonable request, de-identified data can be requested via email to jing_ling_tay@imh.com.sg, for up to 5 years after the publication of this study.

## References

[1] World Health Organisation Child and Adolescent Mental Health. http://www.who.int/mental_health/maternal-child/child_adolescent/en/.

[2] World Health Organization The World Health Report 2001: Mental Disorders Affect One in Four People. https://www.who.int/news/item/28-09-2001-the-world-health-report-2001-mental-disorders-affect-one-in-four-people.

[3] Bu D., Chung P.K., Zhang C.Q., Liu J., Wang X. (2020). Mental health literacy intervention on help-seeking in athletes: A systematic review. Int. J. Environ. Res. Public Health.

[4] Daviu N., Bruchas M.R., Moghaddam B., Sandi C., Beyeler A. (2019). Neurobiological links between stress and anxiety. Neurobiol. Stress.

[5] Lazarus R.S., Folkman S. (1984). Stress, Appraisal, and Coping.

[6] Deci E.L., Ryan R.M. (2008). Self-determination theory: A macrotheory of human motivation, development, and health. Can. Psychol./Psychol. Can..

[7] Ryan R.M., Deci E.L. (2001). On happiness and human potentials: A review of research on hedonic and eudaimonic well-being. Annu. Rev. Psychol..

[8] Ryff C.D. (1989). Happiness is everything, or is it? Explorations on the meaning of psychological well-being. J. Personal. Soc. Psychol..

[9] Browne J., Penn D.L., Meyer-Kalos P.S., Mueser K.T., Estroff S.E., Brunette M.F., Correll C.U., Robinson J., Rosenheck R.A., Schooler N. (2017). Psychological well-being and mental health recovery in the NIMH RAISE early treatment program. Schizophr. Res..

[10] Jorm A.F., Korten A.E., Jacomb P.A., Christensen H., Rodgers B., Pollitt P. (1997). “Mental health literacy”: A survey of the public’s ability to recognise mental disorders and their beliefs about the effectiveness of treatment. Med. J. Aust..

[11] Reavley N.J., Jorm A.F. (2012). Public recognition of mental disorders and beliefs about treatment: Changes in Australia over 16 years. Br. J. Psychiatry.

[12] Sebastian J., Richards D. (2017). Changing stigmatizing attitudes to mental health via education and contact with embodied conversational agents. Comput. Hum. Behav..

[13] Taylor-Rodgers E., Batterham P.J. (2014). Evaluation of an online psychoeducation intervention to promote mental health help seeking attitudes and intentions among young adults: Randomised controlled trial. J. Affect. Disord..

[14] Hendriks T., Schotanus-Dijkstra M., Hassankhan A., De Jong J., Bohlmeijer E. (2020). The efficacy of multi-component positive psychology interventions: A systematic review and meta-analysis of randomized controlled trials. J. Happiness Stud..

[15] Tay J.L., Goh Y.-S.S., Klainin-Yobas P. (2020). Online HOPE intervention on mental health literacy among youths in Singapore: An RCT protocol. J. Adv. Nurs..

[16] AI-Therapy Statistics Sample Size Calculator. https://www.ai-therapy.com/psychology-statistics/sample-size-calculator.

[17] Li T.M., Chau M., Wong P.W., Lai E.S., Yip P.S. (2013). Evaluation of a web-based social network electronic game in enhancing mental health literacy for young people. J. Med. Internet Res..

[18] Tay J.L., Tay Y.F., Klainin-Yobas P. (2018). Effectiveness of information and communication technologies interventions to increase mental health literacy: A systematic review. Early Interv. Psychiatry.

[19] O’Reilly C.L., Bell J.S., Kelly P.J., Chen T.F. (2011). Impact of mental health first aid training on pharmacy students’ knowledge, attitudes and self-reported behaviour: A controlled trial. Aust. N. Z. J. Psychiatry.

[20] Bond K.S., Jorm A.F., Kitchener B.A., Reavley N.J. (2015). Mental health first aid training for Australian medical and nursing students: An evaluation study. BMC Psychol..

[21] Gulliver A., Griffiths K.M., Christensen H., Mackinnon A., Calear A.L., Parsons A., Bennett K., Batterham P.J., Stanimirovic R. (2012). Internet-based interventions to promote mental health help-seeking in elite athletes: An exploratory randomized controlled trial. J. Med. Internet Res..

[22] Ribeiro Z.M.T., Spadella M.A. (2018). Content validation of educational material on healthy eating for children under two years of age. Rev. Paul. Pediatr..

[23] Griffiths K.M., Christensen H., Jorm A.F., Evans K., Groves C. (2004). Effect of web-based depression literacy and cognitive–behavioural therapy interventions on stigmatising attitudes to depression: Randomised controlled trial. Br. J. Psychiatry.

[24] Chong S.A., Abdin E., Picco L., Pang S., Jeyagurunathan A., Vaingankar J.A., Kwok K.W., Subramaniam M. (2016). Recognition of mental disorders among a multiracial population in Southeast Asia. BMC Psychiatry.

[25] Yap M.B., Mackinnon A., Reavley N., Jorm A.F. (2014). The measurement properties of stigmatizing attitudes towards mental disorders: Results from two community surveys. Int. J. Methods Psychiatr. Res..

[26] Subramaniam M., Abdin E., Picco L., Pang S., Shafie S., Vaingankar J., Kwok K., Verma K., Chong S. (2017). Stigma towards people with mental disorders and its components–a perspective from multi-ethnic Singapore. Epidemiol. Psychiatr. Sci..

[27] Tay J.L., Xia X.S., Tan C.L.R., Qu Y., Loh C.-L.J., Lau Y., Klainin-Yobas P. (2018). Evaluating predicting factors of psychological well-being among university and polytechnic students. Singap. Nurs. J..

[28] Cohen S., Kamarck T., Mermelstein R. (1983). A global measure of perceived stress. J. Health Soc. Behav..

[29] Cohen S., Spacapan S., Oskamp S. (1988). Perceived Stress in a Probability Sample of the United States. The Social Psychology of Health.

[30] Teh H.C., Archer J.A., Chang W., Chen S.A. (2015). Mental well-being mediates the relationship between perceived stress and perceived health. Stress Health.

[31] Gillespie-Lynch K., Brooks P.J., Someki F., Obeid R., Shane-Simpson C., Kapp S.K., Daou N., Smith D.S. (2015). Changing college students’ conceptions of autism: An online training to increase knowledge and decrease stigma. J. Autism Dev. Disord..

[32] Reavley N.J., Mackinnon A.J., Morgan A.J., Jorm A.F. (2014). Stigmatising attitudes towards people with mental disorders: A comparison of Australian health professionals with the general community. Aust. N. Z. J. Psychiatry.

[33] Wright A., McGorry P.D., Harris M.G., Jorm A.F., Pennell K. (2006). Development and evaluation of a youth mental health community awareness campaign–The Compass Strategy. BMC Public Health.

[34] Mamun M.A., Naher S., Moonajilin M.S., Jobayar A.M., Rayhan I., Kircaburun K., Griffiths M.D. (2020). Depression literacy and awareness programs among Bangladeshi students: An online survey. Heliyon.

[35] Amarasuriya S.D., Jorm A.F., Reavley N.J. (2015). Quantifying and predicting depression literacy of undergraduates: A cross sectional study in Sri Lanka. BMC Psychiatry.

[36] Coles M.E., Ravid A., Gibb B., George-Denn D., Bronstein L.R., McLeod S. (2016). Adolescent mental health literacy: Young people’s knowledge of depression and social anxiety disorder. J. Adolesc. Health.

[37] Cotton S.M., Wright A., Harris M.G., Jorm A.F., McGorry P.D. (2006). Influence of gender on mental health literacy in young Australians. Aust. N. Z. J. Psychiatry.

[38] Lauber C., Ajdacic-Gross V., Fritschi N., Stulz N., Rössler W. (2005). Mental health literacy in an educational elite–an online survey among university students. BMC Public Health.

[39] Townsend L., Musci R., Stuart E., Heley K., Beaudry M.B., Schweizer B., Ruble A., Swartz K., Wilcox H. (2019). Gender differences in depression literacy and stigma after a randomized controlled evaluation of a universal depression education program. J. Adolesc. Health.

[40] Wang J., Adair C., Fick G., Lai D., Evans B., Perry B.W., Jorm A., Addington D. (2007). Depression literacy in Alberta: Findings from a general population sample. Can. J. Psychiatry.

[41] Lambie J.A., Lindberg A. (2016). The role of maternal emotional validation and invalidation on children’s emotional awareness. Merrill-Palmer Q..

[42] Reavley N.J., McCann T.V., Jorm A.F. (2012). Mental health literacy in higher education students. Early Interv. Psychiatry.

[43] Furnham A., Lousley C. (2013). Mental health literacy and the anxiety disorders. Health.

[44] Paulus D.J., Wadsworth L.P., Hayes-Skelton S.A. (2015). Mental health literacy for anxiety disorders: How perceptions of symptom severity might relate to recognition of psychological distress. J. Public Ment. Health.

[45] Arafat S.Y., Ahmed S., Uddin S. (2018). Depression literacy status in Bangladesh: A cross-sectional comparative observation. J. Behav. Health.

[46] Bhuiyan M.A.H., Griffiths M.D., Mamun M.A. (2020). Depression literacy among Bangladeshi pre-university students: Differences based on gender, educational attainment, depression, and anxiety. Asian J. Psychiatry.

[47] Furnham A., Hamid A. (2014). Mental health literacy in non-western countries: A review of the recent literature. Ment. Health Rev. J..

[48] Tippin G.K., Maranzan K.A. (2019). Efficacy of a Photovoice-based video as an online mental illness anti-stigma intervention and the role of empathy in audience response: A randomized controlled trial. J. Appl. Soc. Psychol..

[49] Nasiri S., Akbari H., Tagharrobi L., Tabatabaee A.S. (2018). The effect of progressive muscle relaxation and guided imagery on stress, anxiety, and depression of pregnant women referred to health centers. J. Educ. Health Promot..

[50] McGillivray J., Evert H. (2014). Group cognitive behavioural therapy program shows potential in reducing symptoms of depression and stress among young people with ASD. J. Autism Dev. Disord..

[51] Sizoo B.B., Kuiper E. (2017). Cognitive behavioural therapy and mindfulness based stress reduction may be equally effective in reducing anxiety and depression in adults with autism spectrum disorders. Res. Dev. Disabil..

[52] Khatamian Far P. (2018). Challenges of recruitment and retention of university students as research participants: Lessons learned from a pilot study. J. Aust. Libr. Inf. Assoc..

